# Deep Learning-Based Multilevel Classification of Alzheimer’s Disease Using Non-invasive Functional Near-Infrared Spectroscopy

**DOI:** 10.3389/fnagi.2022.810125

**Published:** 2022-04-26

**Authors:** Thi Kieu Khanh Ho, Minhee Kim, Younghun Jeon, Byeong C. Kim, Jae Gwan Kim, Kun Ho Lee, Jong-In Song, Jeonghwan Gwak

**Affiliations:** ^1^Department of Software, Korea National University of Transportation, Chungju, South Korea; ^2^Department of Biomedical Science and Engineering, Gwangju Institute of Science and Technology, Gwangju, South Korea; ^3^School of Electrical Engineering and Computer Science, Gwangju Institute of Science and Technology, Gwangju, South Korea; ^4^Department of Neurology, Chonnam National University Medical School, Gwangju, South Korea; ^5^Gwangju Alzheimer’s Disease and Related Dementias Cohort Research Center, Chosun University, Gwangju, South Korea; ^6^Department of Biomedical Science, Chosun University, Gwangju, South Korea; ^7^Korea Brain Research Institute, Daegu, South Korea; ^8^Department of Biomedical Engineering, Korea National University of Transportation, Chungju, South Korea; ^9^Department of AI Robotics Engineering, Korea National University of Transportation, Chungju, South Korea; ^10^Department of IT and Energy Convergence (BK21 FOUR), Korea National University of Transportation, Chungju, South Korea

**Keywords:** Alzheimer’s disease, fNIRS, multi-class classification, deep learning – artificial neural network (DL-ANN), CNN-LSTM

## Abstract

The timely diagnosis of Alzheimer’s disease (AD) and its prodromal stages is critically important for the patients, who manifest different neurodegenerative severity and progression risks, to take intervention and early symptomatic treatments before the brain damage is shaped. As one of the promising techniques, functional near-infrared spectroscopy (fNIRS) has been widely employed to support early-stage AD diagnosis. This study aims to validate the capability of fNIRS coupled with Deep Learning (DL) models for AD multi-class classification. First, a comprehensive experimental design, including the resting, cognitive, memory, and verbal tasks was conducted. Second, to precisely evaluate the AD progression, we thoroughly examined the change of hemodynamic responses measured in the prefrontal cortex among four subject groups and among genders. Then, we adopted a set of DL architectures on an extremely imbalanced fNIRS dataset. The results indicated that the statistical difference between subject groups did exist during memory and verbal tasks. This presented the correlation of the level of hemoglobin activation and the degree of AD severity. There was also a gender effect on the hemoglobin changes due to the functional stimulation in our study. Moreover, we demonstrated the potential of distinguished DL models, which boosted the multi-class classification performance. The highest accuracy was achieved by Convolutional Neural Network-Long Short-Term Memory (CNN-LSTM) using the original dataset of three hemoglobin types (0.909 ± 0.012 on average). Compared to conventional machine learning algorithms, DL models produced a better classification performance. These findings demonstrated the capability of DL frameworks on the imbalanced class distribution analysis and validated the great potential of fNIRS-based approaches to be further contributed to the development of AD diagnosis systems.

## Introduction

The incidence of age-related dementia has increased dramatically as the world population is aging. Dementia was estimated to affect 50 million people worldwide in 2018 and is expected to exceed 150 million people within 30 years (Alzheimer’s Disease International, 2018). Alzheimer’s disease (AD) is one of the most well-known causes of dementia, accounting for nearly two-thirds of all dementia patients (Alzheimer’s Association, 2019). AD is characterized by the progressive impairment of both cognitive and memory abilities due to a consequence of the significant loss of neurons in the nervous system (Veitch et al., 2019) or disruption of nerve cell communications by the presence of extracellular Amyloid-Beta (Aβ) peptide plaques and neurofibrillary tangles (NFT), which typically develop decades before the symptoms manifest (Lowe et al., 2019). Since AD first destroys brain cells related to language and memory brain regions, patients suffer from confusion, memory loss, speech impediment, poor problem-solving skills, and difficulty in daily communication. Then, other brain regions for controlling breathing and heart functionality would deteriorate, eventually leading to death. Patients with AD are generally diagnosed in late stages, by which point existing treatments can only decelerate the speed of cognitive declines. As such, a prompt diagnosis to facilitate proper treatments of preclinical and its prodromal stages is significantly crucial. Besides fundamental exams, such as patient interview, Mini-Mental State Examination (MMSE), and physical and neurobiological exams, reliable and powerful neuroimaging techniques, such as from functional magnetic resonance imaging (fMRI) (Cheng et al., 2019), structural magnetic resonance imaging (sMRI) (Wang et al., 2016), positron-emission tomography (PET) (Alster et al., 2019), electroencephalography (EEG) (Mazaheri et al., 2018), or diffusion tensor imaging (DTI) (Hwang et al., 2019) techniques, are desperately needed to furnish more informative diagnosis approach. However, these techniques usually appeal to the clinical interaction between doctors and patients, whilst a more flexible paradigm of doctor-patient interaction is preferable.

Functional near-infrared spectroscopy (Naseer and Hong, 2015) is a scalp-located and non-invasive technique which measures the neural activity using the concentration of oxygenated (HbO) and deoxygenated (Hb) hemoglobin in the brain by calculating by the blood-oxygen-level-dependent effect (Ferrari and Quaresima, 2012). fNIRS offers a variety of advantages over the aforementioned neuroimaging methods, such as a relatively high temporal resolution, absence of ionizing radiation, low cost, high portability, lightweight implementation, and lower susceptibility to motion artifacts during experiments (Lancia et al., 2018; Ho et al., 2019). Unlike commonly sizeable systems (e.g., fMRI, PET, EEG), which prevent the daily activity acquisition in the brain, fNIRS enables the brain measurement with much less intrusive form factors when performing tasks to not interrupt the actual behavior of participants. In AD studies, specifically, fNIRS has verified its own potential (Zeller et al., 2010; Perpetuini et al., 2017). Araki et al. (2014) presented a significant difference in the cerebral blood flows of patients with AD under the effects of memantine. Hemodynamic responses between healthy controls (HC) and patients with AD were also distinguished (Metzger et al., 2016; Li et al., 2019), whilst mild cognitive impairment (MCI) – an early stage of AD – was differentiated from those in the HC group (Ghafoor et al., 2019). These studies have demonstrated that patients with AD typically exhibit lower levels of activation in specific brain regions compared to the HC group during cognitive tasks. In addition, fNIRS is well operable with EEG in the multimodal integration to reinforce the diagnosis accuracy (Fazli et al., 2012). Nevertheless, these previous studies have solely distinguished AD from HC using cognitive tests, whereas different AD stages remain unknown and the pathological mechanism of AD progression has not yet been thoroughly documented. Therefore, in this study, multiple subject groups were recruited and participated in different evaluation tasks (cognitive, memory, and language) to comprehensively compare and evaluate the capability of fNIRS in AD diagnosis.

Machine learning (ML) techniques have been a fundamental element involved in any computer-aided diagnosis systems for the automated prediction of neurological disorders. A Bayes classifier was applied to determine the utility of the eye-tracker, heat flux and median absolute deviation, electrocardiogram armband, EEG headset, and heart rate monitor (Haapalainen et al., 2010). A Gaussian mixture model was used to measure the mental workload of a mental arithmetic task through eye-tracking data (Chen and Epps, 2013). The capability of multiple learning algorithms, including logistic regression, decision trees, naïve Bayes, 1-nearest neighbor, and multilayer perceptron, was evaluated on heart rate data. Subsequently, ML has gained much attention in fNIRS studies. Support vector machines (SVMs) and adaptive boosting as traditional ML algorithms were utilized to distinguish stress levels from the resting states of subjects during Stroop task experiments (Solovey et al., 2014). Logistic regression and SVMs in mental workload classification were also verified (Benerradi et al., 2019).

Despite being successfully applied on signal domains, extracting and selecting features in appropriate ways are still considered as main drawbacks of conventional ML. The cost of these supervised learning methods increases with respect to parameters. As such, these algorithms require a huge amount of labeled training data. Meanwhile, theoretical and biological arguments have strongly recommended a model which is composed of multilayers of nonlinear processing with few labeled inputs in correspondence with human brain activity. Therefore, deep architectures such as Convolutional Neural Network (CNN) and Recurrent Neural Network (RNN) as two common forms of Artificial Neural Network (ANN) have unsurprisingly come to our top priorities in terms of solving optimization problems, automated feature extractions, and multi-class classification tasks. Deep learning (DL) models have been adopted a diversity of applications in practice. CNNs have drawn extensive attention in image classification using various frameworks, such as VGG-16 (Simonyan and Zisserman, 2014) and Residua-Net (He et al., 2016), while RNNs depict connections between computing units that formulate a directed graph along a sequence, thus is popular in time-series data analyses, such as machine translation, time-series prediction, or speech recognition (Lokesh et al., 2019; Wright et al., 2019).

Although the DL-based approach has earned great success in various computer vision tasks, its potential in AD classification using fNIRS are not substantially achievable. First, various preprocessing steps are required since the amplitude and the length of signals are different from patient to patient. Second, a sufficiently large number of datasets is mandatory to efficiently train a DL model. Thus, either an adequate number of original data or artificial data from augmentation or resampling techniques is required. Third, it is arduous to obtain an efficient conversion approach to express medical signals in the form of carrying pertinent information in CNN. Moreover, feeding inputs to a RNN network and training it with gradient descent algorithms (Ruder, 2016), such as long-term independence, remain challenges. To address these challenges, researchers have recently paid exceptional efforts to develop the 1 Dimensional-Convolutional Neural Network (1D-CNN) which can capture spatial-temporal information of signals and long short-term memory (LSTM), as one type of RNNs, which can train and avoid the so-called vanishing gradient issue. Therefore, it is of great interest and importance to address the aforementioned challenges in our study.

The main contributions of this study are summarized as follows: (1) we presented the comprehensive experimental protocol, including cognitive, memory, and verbal tests to investigate the diverse patterns of AD progression, thereby facilitating the investigation of hemodynamic response differences between the HC group and three stages of patients with AD; (2) we inspected the significant differences of oxygen hemodynamic concentrations between subject groups and between gender; and (3) we demonstrated the potential of fNIRS in AD multi-class classification using four DL architectures.

The remainder of this paper is organized as follows: Section “Related Works” briefly presents the related works concerning AD studies using fNIRS and DL techniques. In section “Materials and Methods,” we describe materials, including the experimental protocols, fNIRS datasets, pre-processing steps, and DL models for multi-class classification tasks in detail. Section “Experimental Results” summarizes the experimental classification results. In section “Conclusions and Future Works,” we conclude the paper with our main contributions and some suggestions for future works.

## Related Works

Functional near-infrared spectroscopy is a non-invasive way of measuring cerebral hemodynamic change using near-infrared rays (Yücel et al., 2017). Irani et al. (2007) proved the relatively superior spatial-resolution of fNIRS compared to EEG/Magnetoencephalography (MEG). fNIRS was used to successfully distinguish signals from nearby measured brain regions, thus avoiding fake correlations as induced by EEG/MEG (Sonkaya, 2018). Moreover, from a practical point of view, fNIRS is portable, safe, quiet, relatively inexpensive, easy to handle, has fewer restrictions on subjects, compatible with iron metals, and feasible for long-term continuous and repeated measurements at short intervals. These advantages outweigh the merits of other neuroimaging techniques in the study of neurological disorders and psychiatric disorders such as dementia and brain-related disorders. Many studies have examined the validity of using fNIRS to compare the hemodynamic response in HC and patients with AD, and demonstrated that patients with AD showed lower activation in specific brain regions during various cognitive tasks than normal people’s (Beishon et al., 2017; Vermeij et al., 2017; Liao et al., 2019). Hock et al. (1997) reported that there was a decrease in HbO and Total Hemoglobin (HbT) given a verbal fluency task (VFT) in the parietal cortex of patients with AD. Arai et al. (2006) revealed that HbO concentrations were significantly reduced in the frontal and bilateral parietal areas of the AD group, whereas the hemoglobin activation was only lower in the right parietal area of the MCI group. These findings suggest that fNIRS holds the promising potential to detect AD, even at early stages.

To monitor the declined cognitive function of AD, it is essential to further define a classification scheme. Recent studies have adopted ML on the classification of AD stages. With the development of computational resources, the application of ML algorithms has enabled the work of AD stage labeling based on a classification model, which is beyond the traditional analysis. Cicalese et al. (2020) presented a linear discriminant analysis (LDA) algorithm applied on a hybrid EEG-fNIRS dataset to classify four subject groups (8 HC, 8 MCI, 6 MAD, and 7 MSAD) given a random digit encoding-retrieval task. Their results indicated that the right prefrontal and left parietal regions were relevant to AD progression. The integrated EEG-fNIRS feature set achieved a higher accuracy (79.31%) compared to using EEG (65.52%) or fNIRS (58.62%) alone. Yang et al. (2018) aimed to investigate MCI assessment using statistical analysis and an LDA algorithm to differentiate MCI from HC. They yielded an effective ML tool for early prediction of AD using digital biomarkers and fNIRS (Yang and Hong, 2019). Despite its popularity, conventional ML algorithms have been criticized for their poor performance on raw data, not being limited to on bio-signal domains, and for the prerequisite step of manually extracting informative features (Plis et al., 2014; LeCun et al., 2015).

The ability to achieve features with higher orders of abstraction and complexity compared to conventional ML, such as SVM, makes DL better suited for detecting scattered, complex, and subtle patterns of the data. In addition, an integral facet of DL to differentiate it from other ML methods is that features are not manually engineered. Instead, DL learns the data in an end-to-end manner, resulting in a more objective and less bias-prone process (Plis et al., 2014). Proven for its superior image classification and recognition capabilities, CNN has motivated the development of a CNN-based taxonomy for early-stage AD detection (Vieira et al., 2017). This review paper identified a total of 25 articles relevant to DL studies of psychiatric and neurological disorders, including AD-related concerns. First, the diagnostic studies aimed to classify patients from HC. Second, studies on conversion to illness used the baseline scan from individuals who were predicted as being at high risk of developing a psychiatric or neurologic abnormality. Finally, studies on predicting the treatment response employed the baseline scan from subjects with a neurological or psychiatric diagnosis to predict the subsequent treatment response. The accuracies of DL architectures from those articles were also reported to testify DL feasibility in discriminating between more than two classes (HC, MCI, and AD).

Oh et al. (2019) presented volumetric CNN-based approaches [convolutional autoencoder (CAE)-based unsupervised learning and supervised transfer learning] for four binary classification tasks (AD vs. HC, progressive MCI vs. HC, stable MCI vs. HC, and progressive MCI vs. stable MCI) and a gradient-based visualization task of the spatial attention. They discovered the important AD-related biomarker using an MRI dataset from Alzheimer’s Disease Neuroimaging Initiative (ADNI) database without human intervention (Oh et al., 2019). The experimental results showed that their proposed models achieved favorable performance and comparable efficiency to current state-of-the-art models. Meanwhile, the multimodal DL approach has exerted to incorporate different types of inputs and DL models to boost the AD classification accuracy. Lee et al. (2019) extracted multiple features from MRI, cohort data, and cerebrospinal fluid (CSF) data and adopted an RNN to predict AD. Suk and Shen (2013) employed MMSE, MRI, PET, and CSF to discriminate AD from MCI. Feng et al. (2019) fed MRI and PET data to the incorporated framework of 3D-CNN and LSTM.

Previous AD studies have validated not only the potential of fNIRS in assisting clinicians/practitioners to distinguish different AD groups from a HC group, but also the potential of DL on AD analysis. However, to the best of our knowledge, there are several issues that remain unsolved and needed to be addressed. The comprehensive experiment design with different tasks to investigate AD patterns and the DL classification frameworks for AD studies using imbalanced fNIRS datasets have not been thoroughly documented. We, therefore, conducted entire experiments (cognitive, memory, and verbal tests) and verified a collection of four DL models to differentiate the HC group from three levels of AD progression using the fNIRS dataset.

## Materials and Methods

### Subjects

Early-phase AD is hardly characterized since it precedes for years as an asymptomatic AD that is not comparable to the normal aging process. However, using neuropathological biomarkers from the cerebrospinal fluid and several brain imaging techniques, the sequence of AD development is distinguishable from that of aging in a cognitively unimpaired healthy state that does not have AD biomarkers. Hence, the National Research Center for Dementia and Chonnam National University Hospital (Gwangju, South Korea) recruited senior citizens living in Gwangju and adjacent cities to approach this objective. By conducting a series of medical examinations (MMSE, PET, MRI, and patient interview), subjects were diagnosed with disease stages according to the guidelines from the National Institute of Neurological and Communicative Disorders and Stroke (NINCDS)-Alzheimer’s Disease and Related Disorders Association (ADRDA) Work Group (McKhann et al., 1984), the National Institute on Aging and Alzheimer’s Association (NIA-AA) (Jack et al., 2011), and International Working Group (IWG) (Cummings et al., 2013). Subjects with a mental and/or behavioral disorder were excluded from this cohort.

The final subject groups included HCs (cognitively normal individuals), asymptomatic AD (abbreviated as aAD: cognitively normal individuals, the amyloid positive in PET was found), prodromal AD (abbreviated as pAD: mild brain dysfunction symptoms such as short-term memory deficits, impaired insight, irritability, dysphoric mood, and anxiety), and AD dementia (abbreviated as ADD: deterioration of memory, language, social abilities that severely effect on daily life) groups. A total of 140 subjects were involved in this study: HC class (*n* = 53, age = 72.7 ± 5.3 years), aAD class (*n* = 28, age = 74.5 ± 4.3 years), pAD class (*n* = 50, age = 75.8 ± 3.9 years), and ADD class (*n* = 9, age = 75.4 ± 6.8 years).

All subjects had no previous experience with our experimental protocol. The purpose of the research and the written consent form were furnished and agreed upon by each subject prior to conducting the experiment. The experimental procedure was approved by the International Review Board at the Gwangju Institute of Science and Technology. The demographic information of participants is summarized in Table 1.

**TABLE 1:** Subject demographics.

	HC	aAD	pAD	ADD
Number	53	28	50	9
Age ± SD (years)	72.7 ± 5.3	74.5 ± 4.3	75.8 ± 3.9	75.4 ± 6.8
Gender (M/F)	21/32	15/13	31/17	4/5
MMSE ± SD	27.0 ± 4.2	26.9 ± 2.5	26.0 ± 3.2	20.2 ± 4.8
Education ± SD	9.8 ± 4.7	10.2 ± 5.2	10.6 ± 5.2	8.5 ± 5.3

### Functional Near-Infrared Spectroscopy Device and Data Preprocessing

**FIGURE 1:**
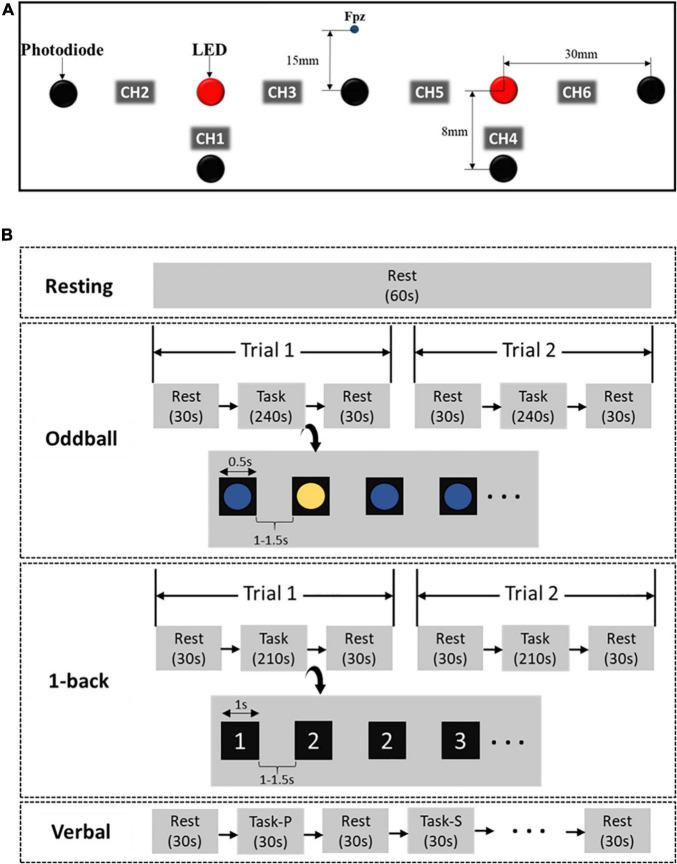
fNIRS device setup **(A)** and experimental protocol **(B)** (s, second; P, phonemic; S, semantic).

Figure 1A represents the fNIRS device setup. fNIRS signals from six channels located in the prefrontal cortex region were recorded. The device was built in the laboratory, and the probe consisted of a pair of source and detector, light-emitting diode (LED; OE-MV7385-P, Opto ENG, South Korea) and photodiodes (Opt101, Texas Instruments). The LED had dual wavelengths (730 and 850 nm). Channels 1, 2, and 3 measured signals in the right prefrontal region while channels 4, 5, and 6 measured signals in the left prefrontal region. While channels 2, 3, 5, and 6 were far-channels (30 mm) and the signals obtained from these channels presented the cerebral hemodynamic response, channels 1 and 4 were known as near-channels since the distance between the source and the detector was very close (8 mm) in order to measure the superficial layer from the head. The photodiode located in between channel 3 and channel 5 was 15 mm distant from the Fpz point in the 10–20 EEG system.

The signal sampling rate was set to 8 Hz. Each channel was visually inspected during the experiment, and channels exhibiting large spikes were marked as noise and excluded from the study. The changes in the concentration of hemoglobin [HbO, Hb, and total hemoglobin (THb)] were computed by the modified Beer–Lambert law (Swinehart, 1962). To remove artifacts, a low-pass filter with a cutoff frequency of 0.5 Hz was applied. Although we utilized four channels in our previous studies (Nguyen et al., 2019) where the near-channels were excluded by employing the extraction algorithm since their function was to get rid of the skin and skull noise from the far-channels, it is crucial to consider inputs from all channels to be fed to proposed DL frameworks in this present study.

### Experimental Protocol

Subjects were requested to sit in a comfortable chair situated in a confined room to reduce environmental disturbances and minimize their body movements during experiments. In our experiment, subjects underwent a resting stage and three brain functional tasks, including Oddball (cognitive ability test), 1-back (memory ability test), and Verbal fluency (language ability test). First, subjects calmly watched a white cross appeared on the monitor screen for 60 s during the resting period. Subjects also had 30 s for resting in a similar way before and after each experimental task.

Second, the blue and yellow circles appeared alternately, and subjects were asked to report the color of each ball in the Oddball task. The yellow circle rarely appeared compared to the blue one. Subjects were asked to pay attention to the occurrence of the circle and press “0” if the yellow circle presented. Each colored circle appeared for 0.5 s, and a blank screen appeared for 1–1.5 s between the circles. The task took 5 min and was recorded twice.

Third, a consecutive series of numbers, from 1 to 3, randomly appeared on the screen in the 1-back task. The appearance of each number lasted for only a second. Subjects were requested to remember a number which was selected and previously presented in one trial. Subjects then pressed the “0” button likewise. The task took 5 min and was also recorded twice.

Lastly, a total of six Korean phonemic and semantic words alternately appeared on the screen in the Verbal fluency task. Initially, a letter appeared alone (i.e., “a”), and subjects would continuously recite complete words that start with the letter (i.e., “anniversary”). If a white cross appeared on the screen, they would stop speaking and carefully glare at the screen until the next semantic word appeared. When the semantic word (i.e., “animal”) appeared, the subjects would continuously recite certain animals (i.e., “Lion”). Each trial block took for 30 s, and there was a 30-s resting state between trials. This process was repeatedly conducted, and the Verbal task took approximately 7 min.

In total, every subject performed six sub-tasks in the order of Resting, Oddball (trial 1), Oddball (trial 2), 1-back (trial 1), 1-back (trial 2), and Verbal fluency. All procedures took around 30 min, including the break time between tasks (see Figure 1B). Any subject who was not able to fulfill any of these sub-tasks was excluded from our analysis.

### Proposed Deep Learning Models

As a subcategory of ML, DL is inspired by the structure and function of the brain, the so-called biological neural networks (NNs). A DL model is made of the basic building block of NNs which are trained on the large-scale labeled dataset(s) to learn functions from the original data without manually extracting features, as most of the conventional ML algorithms do. CNN, denoted as one of the most well-known forms of DL, has been demonstrating its robust performances in the computer vision community where the inputs usually include high dimensional features (i.e., each pixel of an image is a dimension). Hence, it is pertinent to perform on a long sequence of data and is easy to implement. Meanwhile, employing RNN, which can handle the temporal data through modeling the underlying dynamic behaviors of time series sequences, is natural. However, since the sequence data is generated from electrodes that are closely located, there is a strong correlation in time within the sequence. Directly learning sequences with noises from RNNs is very challenging. LSTM or the recently invented Generated Recurrent Units (GRUs) can settle the vanishing or explosion of gradient loss. Due to the early-stopping strategy used during the training process, it is certainly expected that no severe overfitting can be observed. Lastly, to take synergetic advantage of CNN and LSTM, a mixed model including CNN followed by LSTM, where CNN acts as an effective decoder for LSTM layers, is seemingly interesting to build.

#### 1D-Convolutional Neural Network

Since the input size of fNIRS data is different from acquainted image datasets with more than one dimension, a 1D-CNN is necessarily done in the time domain. In this work, with the idea of using 2 dimensional stacks for time-specific activity, the CNN model was employed to decompose and feed-forward a 1D fNIRS signal that was known as a precomputed spectrogram or time-frequency. CNN consisted of one 1D convolutional layer, one 1D max-pooling layer, and two linear layers. Batch normalization was used before the activation function, and dropout was used for regularization. A grid search of convolutional filter number, filter size, pooling size, batch size, number of units per layer, stride, and learning rate was done to optimize hyper-parameters, as shown in Supplementary Table 1.

#### Long Short-Term Memory

To discriminate the subtle spatial-temporal change in the fNIRS feature space associated with different progressive stages of AD, a solution capable of remembering and ultimately aggregating transitions across the dataset is required. LSTM, with the attention mechanism, has been known as a wide-ranging method utilized for distinctly learning and classifying bio-signal time-series datasets (Tsiouris et al., 2018; Zhang et al., 2018, 2019) on fNIRS (Yoo et al., 2018; Sirpal et al., 2019). Thus, it triggered us to apply LSTM to improve the classification performance using fNIRS by focusing on the crucial task-relevant features from different time-steps. Compared to classic approaches in such aforementioned classifiers, RNN or LSTM particularly requires no or almost no feature engineering that an fNIRS dataset is able to feed directly into the network. We utilized the “many to one” architecture, which means using time series of feature vectors (one vector per time step) to convert them to a probability matrix at the output layer.

**FIGURE 2:**
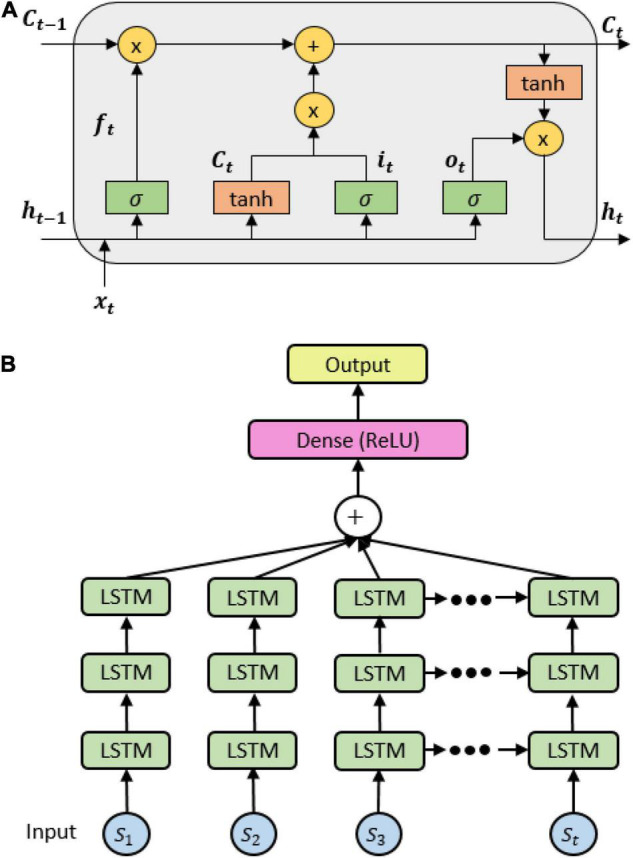
**(A)** A long short-term memory (LSTM) cell internal mechanism; **(B)** the stacked LSTM architecture.

An LSTM network consists of cells whose outputs evolve through the network based on the content of past memory. The cell has a common cell state, keeping long-term dependences along the entire LSTM chain. The flow information is then monitored by the input gate *i_t_* and the forget gate *f_t_*, hence allowing the network to decide whether to forget the previous state *C*_*t–1*_ or to update the current state *C_t_* with new information. The output of each cell, namely, hidden state, is controlled by an output gate *o_t_* that allows the cell to compute its output given the updated cell state (see Figure 2A). These states of an LSTM model are formulated by the following formula:


(1) $$i_{t}=\sigma \left(W_{i}\cdot \left[h_{t-1},x_{t}\right]+b_{i}\right),$$

(2) $$f_{t}=\sigma \left(W_{f}\cdot \left[h_{t-1},x_{t}\right]+b_{f}\right),$$

(3) $$C_{t}=f_{t}*C_{t-1}+i_{t}*tanh\left(W_{c}\cdot \left[h_{t-1},x_{t}\right]+b_{c}\right),$$

(4) $$o_{t}=\sigma \left(W_{o}\cdot \left[h_{t-1},x_{t}\right]+b_{o}\right),$$

(5) $$h_{t}=o_{t}*tanh\left(C_{t}\right),$$

where $\sigma \left(x\right)=\frac{1}{1+e^{-x}}$, *x_t_* is the input features fed into the cell, *h_t_* is the hidden state at time step t, *C*_*t–1*_ is the cell state at time step t, *W_i_*, *W_f_*, *W_c_*, *W_o_* are the weights, and *b_i_*, *b_f_*, *b_c_*, *b_o_* are the biases obtained during the backpropagation process. We thereby proposed an LSTM architecture with three stacked layers and a fully connected layer with rectified linear units (ReLU) function to predict the probability of each class (see Figure 2B).

Several hyper-parameters for the LSTM network were thoroughly explored and tuned to earn the best classification results. Concretely, these hyper-parameters encompassed the recurrent depth, LSTM hidden layer size, batch size, and training epochs. Three stacked LSTM layers (*D*_0_, *D*_1_, *D*_2_) and the weight matrix *L_2_* regularization coefficient was applied for each LSTM layer. In addition, other variables such as learning rate (*l_r_*) and the amount of lambda loss were used for stochastic Adam optimizer. Supplementary Table 2 presents the optimal values for these parameters.

#### Gated Recurrent Units

**FIGURE 3:**
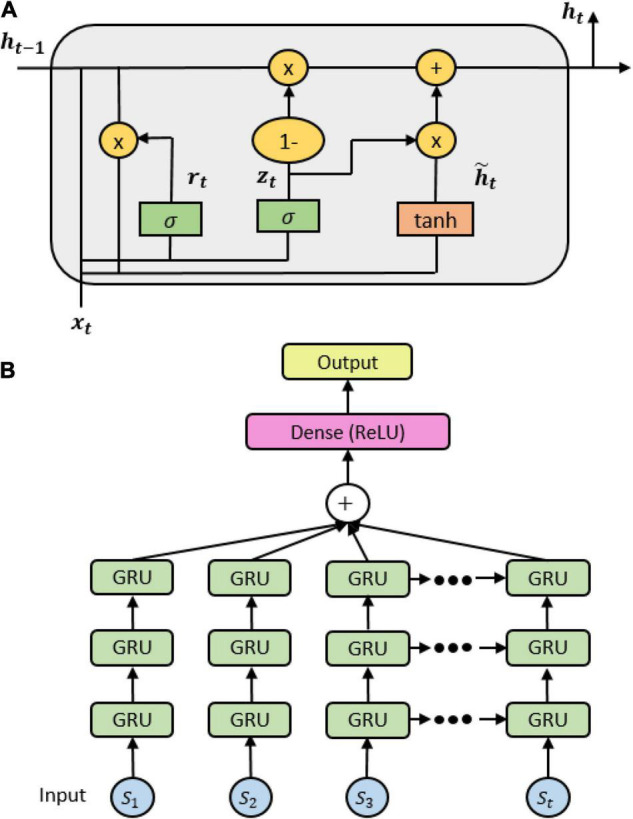
**(A)** A gated recurrent unit (GRU) cell internal mechanism; **(B)** the stacked GRU architecture.

As similar to the precedent LSTM, gated recurrent units (GRU) is newly created for the solution of short-term memory. An internal mechanism called gates is also needed to regulate the flow of information. However, GRU gets rid of the cell state and uses the hidden state to transfer information. It includes only two gates: a reset gate which is another gate used to decide how much past information can be forgotten, and an update gate which acts similar to the forget and input gate of LSTM which decides what information can be discarded and what new information can be complemented (see Figure 3A). These states of a GRU model are formulated by following formulas:


(6) $$z_{t}=\sigma \left(W_{z}\cdot \left[h_{t-1},x_{t}\right]\right)$$

(7) $$r_{t}=\sigma \left(W_{r}\cdot \left[h_{t-1},x_{t}\right]\right)$$

(8) $${\tilde{h}}_{t}=tanh\left(W\cdot \left[r_{t}*h_{6}t-1,x_{t}\right]\right),$$

(9) $$h_{t}=\left(1-z_{t}\right)*h_{t-1}+z_{t}*{\tilde{h}}_{t})$$

Due to its fewer tensor operations, GRU trains data faster than LSTM. It also has a faster convergence, but it is unlikely to guarantee which one outperforms others. In practice, we built a GRU model with the same architecture of LSTM, excluding the module used in the current layers. Three GRU layers were stacked, followed by a fully connected layer and an output layer (see Figure 3B). Batch-normalization and the ReLU activation function were applied in the fully connected layer. The output layer was computed by the cross-entropy loss with a soft-max layer. More details of chosen parameters are depicted in Supplementary Table 3.

#### A Mixed Model: Long Short-Term Memory Model Coupled With Convolutional Neural Network Decoder

Inspired by the phenomenal performance of the hybrid CNN-RNN in both the spatial domain (Dutta et al., 2018) and the spatial-temporal domain (Yang et al., 2018; Shi et al., 2019), we employed a CNN layer as a decoder for original input data, followed by multiple LSTM layers to cope with the decoded temporal data, and a fully connected layer where the cross-entropy loss with a soft-max function was computed for classification (see Supplementary Table 4). We let a CNN layer extract multiple high-level features from the raw data by using a substantially large filter size and stride. The sequence was thus decoded into multiple shorter sequences symbolizing different high-level temporal features.

## Experimental Results

### Statistical Analysis: The Change of Oxygen Hemoglobin Concentrations

**FIGURE 4:**
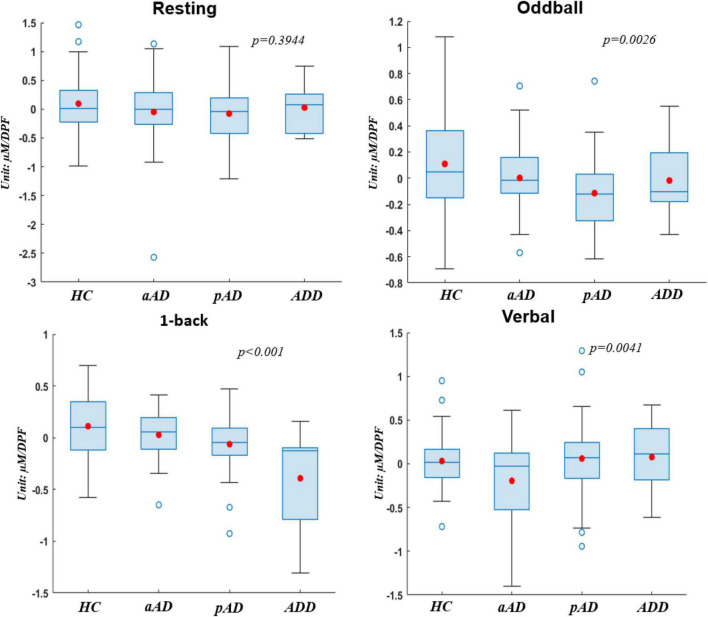
Box charts of oxyhemoglobin (HbO) concentrations acquired by four subject groups under four conditions.

To clearly evaluate the change of hemoglobin concentrations among four subject groups who underwent four experimental stages, we utilized the utmost distinguishable observation from HbO over other hemoglobin types of reduced hemoglobin (HbR) and THb in this part. Figure 4 presents the box chart distributions of HbO obtained from each experimental protocol to compare HC with each level of AD severity. Note that the typical hemodynamic response of brain activation is accompanied by an increase of HbO concentrations (Mangrum et al., 2018). On each box, the central mark is median, the red filled circle is the mean, the edges of each box are the 25th and 75th percentiles, the whiskers denote the standard error of the mean, and the empty blue circle show outliners. All statistical analyses were performed with a significant level at 0.05 as the cutoff of significance, and each box chart was given a *p*-value.

Regarding the difference among subject groups, all groups did not show significant differences (*p* > 0.05) during the resting period, whereas there existed significant differences during the other three experimental tasks (*p* < 0.05). The HC group (values were greater than zero) consistently presented much higher hemodynamic activations than other groups. This indicates that normal people had the highest response and had no difficulty when they performed all the brain ability tasks. Meanwhile, on the second place of AD severity level, pAD patients showed lower values than HC and aAD during resting, oddball, and 1-back tasks. This verifies that pAD patients noted lower activations of hemodynamic responses compared with HC and patients with aAD in the resting period, cognitive ability, and memory ability tests. More interestingly, the significantly statistical difference between subject groups did exist during the 1-back task (*p* < 0.001). Each group behaved and responded differently in this memory task, and their response was perfectly correlated with the degree of AD severity. ADD patients, in particular, denoted the lowest hemodynamic activation, followed by pAD, aAD, and HC.

**FIGURE 5:**
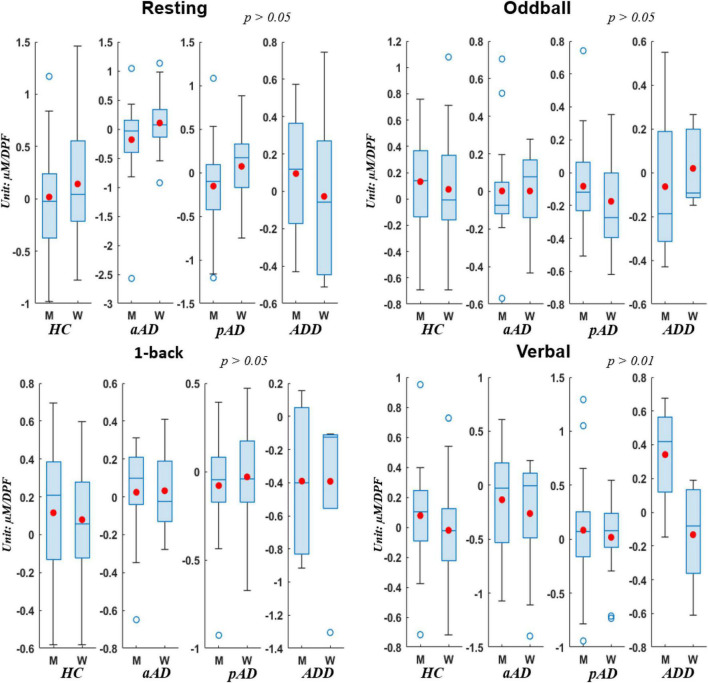
Box charts of HbO concentrations acquired by Men (M) and Women (W) of four subject groups under four conditions.

In addition, we testified the change of HbO concentrations via box chart distributions for all conditions when subjects were sub-grouped by gender (see Figure 5). In general, there was no significant difference between men and women who underwent all three conditions (*p* > 0.05 for resting, oddball, and 1-back stages) whilst there existed relatively important disparity among gender in the verbal stage. Further, men from all four subject groups presented much higher levels of hemodynamic activations than women in most conditions except the resting period in which men from HC, aAD, and pAD groups showed lower hemodynamic activations. This finding suggested that the frontal changes in HbO concentrations during cognitive, memory, and verbal tasks were stronger in men compared to women. Hence, there was a gender effect on relative hemoglobin changes due to the functional stimulation in our study that achieved similar observations from previous studies on evaluating hemodynamic activations based on gender inclusion criteria (Shoemaker et al., 2001; Auger et al., 2016).

### Classification Performance of Deep Learning Models

To precisely validate the potential of deep learning approaches, the original dataset (three hemoglobin types) was used. Four models were built, including 1D-CNN, stacked LSTM, stacked GRU, and a mixed CNN-LSTM. To enhance the classification performance, we segmented the training signals into nonoverlapping fragments with a specified window length. Each split window was assigned a label associated with its ground truth. Then, we ran eight classifier algorithms to differentiate the HC group from the three levels of AD severity. DL models were then adopted to differentiate the HC group from the three levels of AD severity. Five-fold cross-validation was used to evaluate the model’s performance using accuracy-based measurements (Accuracy, Precision, Recall, and F1-score).

In regard to the efficacy of the DL models, all models achieved average accuracies above 80% using all three types of hemoglobin (i.e., accuracies of 82.9, 84.4, 78.1, and 86.8% with HbO type are associated with the performance of 1D-CNN, LSTM, GRU, and CNN-LSTM, respectively). Based on our observations, although LSTM and GRU models had similar concepts of preventing the vanishing gradient problem, their performance in AD multi-classification using fNIRS were exposed differently. GRU was simpler with only two gates, was trained faster, and more computationally efficient, but GRU obtained much lower accuracies than LSTM on all three hemoglobin classes. This indicates that LSTM could outperform GRU since LSTM resembled the theory of remembering longer sequences with a memory unit. This made LSTM more sophisticated and able to produce the network stability in dealing with the fNIRS task, which requires modeling the long-distance relation. In addition, LSTM achieved a higher classification accuracy than 1D-CNN. That is, LSTM, which included less feature compatibility, handled arbitrary input/output lengths, and used its internal memory and time-series information to process the arbitrary sequences of the input, was well-suited for the fNIRS temporal data compared to 1D-CNN.

More importantly, CNN-LSTM outperformed others (86.8, 86.4, and 87.7% corresponding to HbO, HbR, and THb, respectively). CNN and LSTM were not mutually exclusive since either of them could perform image and time-series classification tasks. The model integration could yield an opportunity to bond the two network types to facilitate effectiveness. A CNN alone would be unable to process fNIRS as the input to be classified as it is visually complicated with the temporal characteristic. Meanwhile, the high-level features that were extracted from long sequences of fNIRS signals by CNN were considerably easy for LSTM to deal with due to its shorter lengths. In detail, the highest accuracy averaged among three hemoglobin types was 87.7% and could occasionally peak to values up to 88.9% at some moments of luck during the training process. This relied on how LSTM weights got initialized at the beginning of training. This means that LSTM was able to correctly identify the movement type using decoded CNN features. Thus, CNN-LSTM was more robust in classifying HC with three different AD stages compared to 1D-CNN and LSTM alone. Table 2 generally denotes the robust performance of the DL model of CNN-LSTM on all three hemoglobin types with higher means in terms of different classification metrics than those obtained by other DL models. Regarding the effects of subject group types, since we had an extremely imbalanced dataset (for all three types of hemoglobin concentrations), it is predictable that DL models could easily classify the majority classes (i.e., HC and pAD). As a result, we obtained accuracies with higher means and lower SD on HC and pAD, while groups of patients with aAD and patients with ADD achieved lower classification accuracies.

**TABLE 2:** Comparison of classification metric results as shown by four proposed deep learning (DL) models using original data.

Class	Accuracy			Precision			Recall			F1-Score		
	HbO	HbR	THb	HbO	HbR	THb	HbO	HbR	THb	HbO	HbR	THb

**1D-Convolutional Neural Network (1D-CNN)**												
HC	0.863	0.853	0.866	0.884	0.862	0.870	0.863	0.853	0.866	0.848	0.852	0.862
aAD	0.826	0.817	0.779	0.847	0.879	0.819	0.826	0.817	0.779	0.806	0.810	0.774
pAD	0.854	0.861	0.876	0.878	0.842	0.912	0.854	0.861	0.876	0.849	0.860	0.875
ADD	0.774	0.731	0.786	0.778	0.864	0.772	0.754	0.711	0.736	0.749	0.710	0.735
Mean	0.829	0.815	0.827	0.847	0.862	0.843	0.824	0.810	0.814	0.813	0.808	0.811

**Long Short-term memory (LSTM)**												

HC	0.897	0.855	0.875	0.913	0.835	0.903	0.897	0.855	0.875	0.893	0.850	0.874
aAD	0.830	0.836	0.848	0.904	0.907	0.927	0.830	0.836	0.848	0.837	0.835	0.836
pAD	0.867	0.863	0.879	0.887	0.900	0.925	0.867	0.863	0.879	0.858	0.874	0.872
ADD	0.781	0.761	0.795	0.764	0.762	0.747	0.751	0.731	0.735	0.751	0.721	0.732
Mean	0.844	0.829	0.849	0.867	0.851	0.875	0.836	0.821	0.834	0.835	0.820	0.828

**Gated Recurrent Units (GRUs)**												

HC	0.816	0.765	0.771	0.839	0.850	0.810	0.816	0.765	0.771	0.812	0.760	0.774
aAD	0.792	0.727	0.741	0.811	0.808	0.785	0.792	0.727	0.741	0.770	0.719	0.739
pAD	0.811	0.779	0.805	0.837	0.868	0.849	0.811	0.779	0.805	0.808	0.778	0.805
ADD	0.705	0.695	0.705	0.721	0.764	0.736	0.705	0.695	0.705	0.706	0.697	0.708
Mean	0.781	0.742	0.756	0.802	0.823	0.795	0.781	0.742	0.756	0.774	0.738	0.756

**CNN-LSTM**												

HC	0.918	0.907	0.917	0.938	0.905	0.918	0.918	0.907	0.917	0.898	0.902	0.913
aAD	0.879	0.855	0.889	0.865	0.872	0.917	0.839	0.855	0.859	0.835	0.853	0.864
pAD	0.880	0.893	0.907	0.902	0.890	0.910	0.880	0.893	0.907	0.876	0.894	0.907
ADD	0.797	0.799	0.793	0.781	0.839	0.771	0.767	0.799	0.753	0.767	0.806	0.746
Mean	0.868	0.864	0.877	0.871	0.877	0.879	0.851	0.864	0.859	0.844	0.864	0.858

**FIGURE 6:**
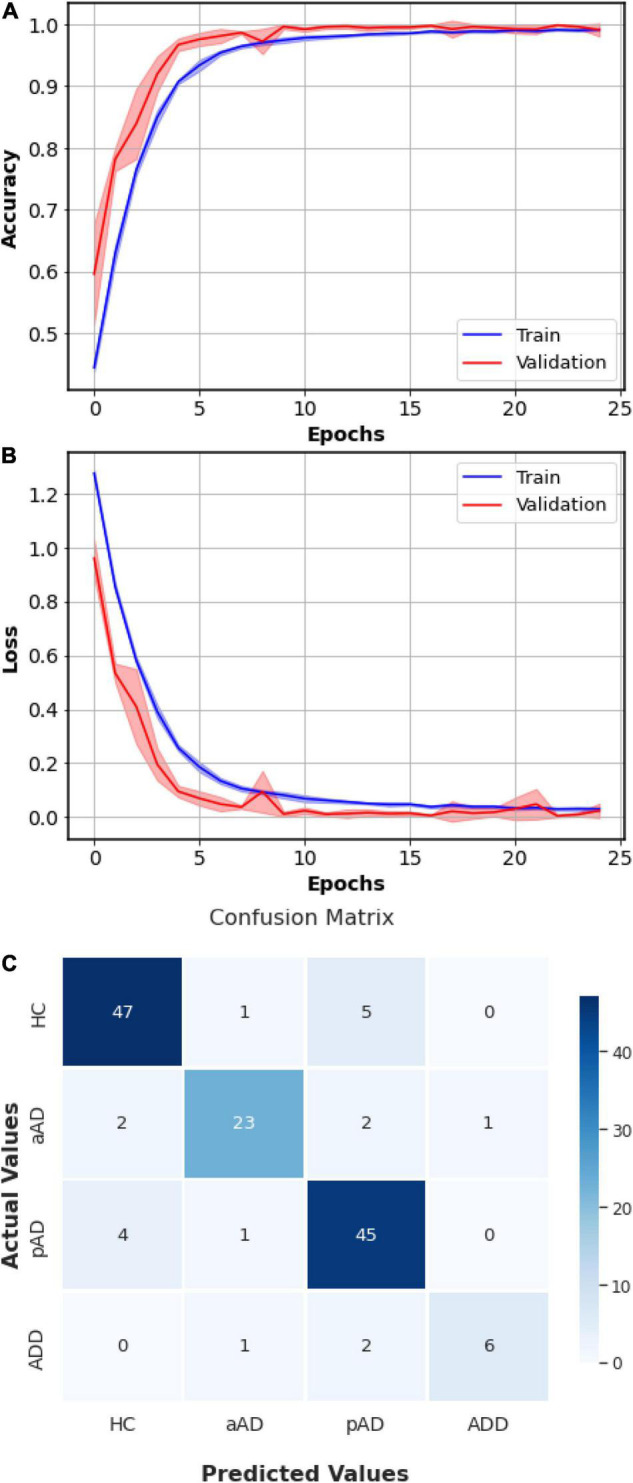
Summarized history of Cognitive Neural Network-Long Short-Term Memory (CNN-LSTM) model’s accuracies **(A)** and losses **(B)** and conclusive confusion matrix **(C)** of the fivefold cross validation. In the accuracy and loss curves, the solid lines indicate the mean and the shadow areas represent the ranges over the fivefolds.

The training’s session progress of CNN-LSTM, including Training and Validation accuracies (Figure 6A and losses Figure 6B), is presented. Initially, the validation loss was slightly similar or lower than the training loss. As long as the validation loss was higher, we would stop the training. In addition, both training and validation accuracies should approach to 1. This demonstrates that the training of CNN-LSTM was exposed to have a stable and accurate performance. As shown in Figure 6C, the overall confusion matrix which was obtained visualized the prediction accuracy of CNN-LSTM by comparing the actual and predicted classes. The overall confusion matrix was produced by the full set of actual and predicted classes to visualize the performance and effectiveness of the algorithms. In particular, for each of five splits, we fitted the training data to the model, and the fitted model was used to predict the classes in the current fold. All actual classes and predicted classes were then appended after fivefolds. The model nearly predicted HC correctly (six misclassified samples), aAD (five misclassified samples), pAD class (five misclassified samples), and ADD (three misclassified samples) in the total of 140 patients.

### Comparison of the Performance of Deep Learning-Based Approaches With Traditional Machine Learning Classifiers

To empirically verify the potential of DL techniques in AD classification using fNIRS, we compared them with the performances of seven conventional ML algorithms, including:

• Linear Discriminant Analysis (LDA) (Li et al., 2006): A set of *n* samples {*x*_1_, *x*_2_, …, *x*_*n*_}was assigned to four AD classes. We calculated the intra-class *M*_*intra*_ and inter-class *M*_*inter*_ matrices samples (discriminant features) and performed the classification task on the transformed space using the Euclidean distance with the 200 epochs training. LDA offered an elegant way to reduce dimension and to possibly classify four classes using those discriminant features.
• K-Nearest Neighbors (KNN) (Zhang and Zhou, 2007): *K* = 4 (number of clusters) was assigned as the most optimal value. The KNN algorithm computed the distance between each data point and its cluster centroid and minimized the error. The point located at a minimum distance from the testing point was supposed to belong to the same class. After 200 epochs, KNN could capture the distant proximity for four class-classification. However, its performance gradually slowed down as the number of data points increased.
• Gaussian Naive Bayes (GNB) (Griffis et al., 2016): We computed the prior *P*(*c*) and posterior probability *P*(*x*|*c*). Then, the conditional probability for each class (given a test sample) was calculated *P*(*c*_*i*_|*x*) based on the Naive Bayes (NB) theory. The class presenting the highest probability among the four classes was predicted as the target class. GNB was the simplest and easiest algorithm among all conventional ML methods.
• Support Vector Machine (SVM) (Lee and Lee, 2007): To non-linearly separate four classes, SVM was applied to construct the decision hyperplanes whereby the margin of the classifier was maximized. The sigmoid kernel trick was employed to do the data transformation and seek optimal boundaries between classes, although this mimicked the idea of the two-layer perception and generated less errors when dealing with non-linear fNIRS data. The number of epochs was not assigned since SVM would stop training when the margin error was very trivial. In addition, no further optimization was required. The time-consuming and computationally intensive resources were more demanding compared to previous classifiers.
• Adaptive Boosting (AdaBoost) (Hastie et al., 2009): A weak classifier was initially built to generate class labels using unweighted training samples. If any misclassified data point was found during training, the weight of that training point was boosted and updated to the next classifier. The procedure was repeated while each classifier had its own score. The final classifier was defined by the linear combination of 1,000 weak classifiers to boost accuracy. The number of estimators was 200 to control the boosting process, and the higher amount of training time was inevitable.
• Random Forest (RF) (Gray et al., 2013): Starting with the selection of arbitrary samples from the given dataset, a decision tree was built for each sample and produced predicted classes. RF consisted of multiple decision trees that would vote for each class. The class was then selected based on the most votes to be the final prediction. The max depth of the tree was set as 8 and the number of estimators was 200. RF was trained several times, and the random state was fixed to guarantee the same sequence of samples and to achieve a deterministic behavior.
• Ensemble Learning (Kang et al., 2015): Six base classifiers (aforementioned algorithms) were integrated to yield a strong and diverse set of base learners to find the optimal combination. By selecting features from these weak learners via a meta-classifier, it was not easily vulnerable to overfitting. We predicted the final class label using the hard (majority) voting method. The result was explicitly equal or better than the best of base classifiers although, it costed longer training time and features. demanding memory.
• Neural Network (NN) (Amato et al., 2013): Compared to previous ML algorithms, NN could learn non-linear and complex relationships of inputs and outputs to constitute better generalization and classification accuracies. After training NN with different settings of hyper-parameters, we came up with the final set of neurons: an input layer, the set of hidden neurons (100-80-50) organized in the form of three hidden layers, and an output layer (four neurons). We manually selected a ReLU activation function, Adam optimizer, 1e-5 learning rate, and 500-iteration duration. NN was relatively hard to train and required a large number of parameters.

**FIGURE 7:**
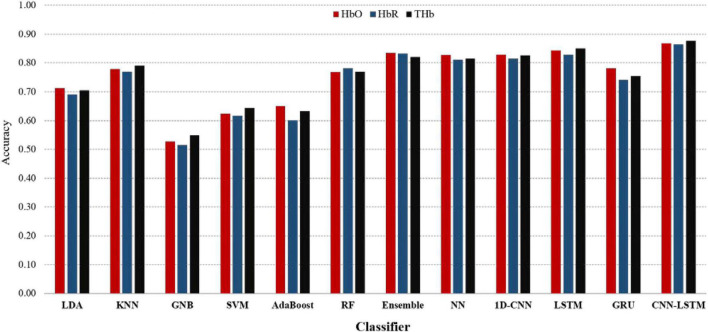
Classification comparisons of accuracy between eight machine learning (ML) classifiers and four DL models using original HB datasets.

As presented in Figure 7, among traditional ML algorithms, the highest accuracy was obtained by Ensemble (82.9% in average), followed by NN, KNN, RF, LDA, SVM, AdaBoost, and GNB (81.8, 77.9, 77.2, 70.3, 63.9, 62.7, and 53.0%, respectively). This indicates that NN was substantially flexible to adopt our adequate data size without considering any feature-engineering steps or structured data and was able to learn high-level features in an end-to-end manner. Meanwhile, most of the features to be identified (to reduce the complexity), split into different parts (to make patterns more tangible), and recombined in the final stage were all preliminary steps for other ML classifiers. However, the obtained result was somewhat poor. In contrast, DL or CNN-LSTM, in particular, validated its robust performance to scale the accuracy up to 87.7%. The results revealed that CNN-LSTM obtained the highest accuracies, tallying with three hemoglobin types, followed by LSTM, NN, 1D-CNN, and Ensemble. In general, the DL algorithms outperform the ML algorithms. Among them, CNN-LSTM elevates accuracy to 3% compared to NN (as the best performance of the ML algorithms).

## Conclusion and Future Works

Developing an approach to identify individuals before or during the earliest stages of AD, with the hope that the urgent intervention could significantly prevent the onset of clinical symptoms, is desperately needed. Due to somewhat similar symptomologies shared by moderate AD with other neurological disorders, the difficulty of monitoring AD progression that results in diagnostic uncertainties and the subsequent suspension of prompt treatments still remains. Therefore, this present study, on one hand, aimed to present the potential capability of fNIRS in AD analysis. A cohort of 140 participants ranging from HC to three severity levels of patients with AD was included. We verified that there were significant oscillations of hemoglobin concentrations recorded from the prefrontal cortex regions among four subject groups and among gender using an intensive experimental design (cognitive, memory, and verbal fluency tests). On the other hand, a collection of DL architectures demonstrated its potential application to fundamentally advance the search for AD multi-classification using fNIRS signals. Three groups of pathological patients with AD were remarkably distinguished from HC with the top highest accuracy of 90.91 and 90.04% achieved by CNN-LSTM and LSTM, respectively. More importantly, DL could better learn complex and abstract representations of an imbalanced fNIRS dataset and presented its outperformance compared to seven traditional ML classifiers in AD analysis. These findings, thereby, furnish preliminary evidence supporting the potential roles of fNIRS coupled with DL to accurately diagnose and assess the AD severity in future development.

Although this study has yielded the feasibility of utilizing different DL models on the fNIRS dataset to discriminate patients at diverse AD stages, several improvements would be required for the future works. First, it is critically crucial to represent the property of hemodynamic responses of three hemoglobin types of fNIRS signals, such as variance, kurtosis, skewness, or initial dips, to precisely quantify the difference among four groups. Second, since the DL performance counted on the relatively small sample size, which could easily lead to the misclassification problem, either a larger cohort or advanced augmentation techniques used for bio-signal domains should be carried out to extend our present findings. Lastly, although CNN-LSTM was very powerful to result in competitive classification performance, other advanced DL architectures should be developed.

## Data Availability Statement

The datasets presented in this article are not readily available because the research was conducted with the research support of the National Research Foundation of Korea and Gwangju Metropolitan City, and approval from the relevant institutions is required. Requests to access the datasets should be directed to JG, james.han.gwak@gmail.com; jgwak@ut.ac.kr.

## Ethics Statement

The studies involving human participants were reviewed and approved by IRB Committee of Gwangju Institute of Science and Technology (from IRB No. 20180629-HR-36-07-01 to 20180629-HR-36-07-05). The patients/participants provided their written informed consent to participate in this study.

## Author Contributions

TH and JG designed the study and wrote the original draft. MK, JK, and JG designed the experimental protocol. MK and JK designed the NIRS system. BK, KL, and J-IS contributed to the collection of patient samples and related data. MK conducted fNIRS data preprocessing steps. TH conducted the experiments and analyzed the obtained results. JG edited the final manuscript. All authors reviewed the manuscript and gave final approval for the manuscript submission.

## Conflict of Interest

The authors declare that the research was conducted in the absence of any commercial or financial relationships that could be construed as a potential conflict of interest.

## Publisher’s Note

All claims expressed in this article are solely those of the authors and do not necessarily represent those of their affiliated organizations, or those of the publisher, the editors and the reviewers. Any product that may be evaluated in this article, or claim that may be made by its manufacturer, is not guaranteed or endorsed by the publisher.
