# Supplementary material for: Deep Learning-Based Multilevel Classification of Alzheimer’s Disease Using Non-invasive Functional Near-Infrared Spectroscopy
Source: Front Aging Neurosci. 2022 Apr 26;14:810125. doi: 10.3389/fnagi.2022.810125 (PMC9087351; doi:10.3389/fnagi.2022.810125)
Supplement: Supplementary file 1 [file Table_1.docx]

# Appendix

**Supplementary Table 1**

LAYERS AND TRAINING HYPER-PARAMETERS OF 1D-CNN

| **Layer** | **Hyper-parameters** |
| --- | --- |
| Conv1d | filter_num=20, filter_size=12, stride=2 |
| Batchnorm | -- |
| Activation | ReLu |
| Dropout | P=0.5 |
| MaxPool | pool_size=2, stride=2 |
| Flattern | -- |
| Linear | num_unit=20 |
| Activation | ReLu |
| Linear | num_unit=4 |
| Activation | Softmax |

1. Shuffling with the same random seed to ban the impact of data recording order
2. Mean-subtraction to normalize data from different trials

*Optimizer*: Adam; *Loss function*: CrossEntropyLoss; *Learning Rate:* ${10}^{-3}$

**Supplementary Table 2**

LAYERS AND TRAINING HYPER-PARAMETERS OF LSTM

| **Layer** | **Hyper-parameters** |
| --- | --- |
| LSTM | unit_num=40, return_sequences=True |
| Dropout | p=0.6, recurrent_p=0.6 |
| Activation | tanh(output), sigmoid(recurrent) |
| LSTM | unit_num=30, return_sequences=True |
| Dropout | p=0.5, recurrent_p=0.5 |
| Activation | tanh(output), sigmoid(recurrent) |
| LSTM | unit_num=20, return_sequences=True |
| Dropout | p=0.4, recurrent_p=0.4 |
| Activation | tanh(output), sigmoid(recurrent) |
| Linear | num_unit=20 |
| Batchnorm | -- |
| Activation | ReLu |
| Dropout | p=0.5 |
| Linear | num_unit=4 |
| Activation | Softmax |

1. Shuffling with the same random seed to ban the impact of data recording order
2. Mean-subtraction to normalize data from different trials

*Optimizer*: Adam; *Loss function*: CrossEntropyLoss

**Supplementary Table 3**

LAYERS AND TRAINING HYPER-PARAMETERS OF GRU

| **Layer** | **Hyper-parameters** |
| --- | --- |
| GRU | unit_num=40, return_sequences=True |
| Dropout | p=0.6, recurrent_p=0.6 |
| Activation | tanh(output), sigmoid(recurrent) |
| GRU | unit_num=30, return_sequences=True |
| Dropout | p=0.5, recurrent_p=0.5 |
| Activation | tanh(output), sigmoid(recurrent) |
| GRU | unit_num=20, return_sequences=True |
| Dropout | p=0.4, recurrent_p=0.4 |
| Activation | tanh(output), sigmoid(recurrent) |
| Linear | num_unit=20 |
| Batchnorm | -- |
| Activation | ReLu |
| Dropout | p=0.5 |
| Linear | num_unit=4 |
| Activation | Softmax |

1. Shuffling with the same random seed to ban the impact of data recording order
2. Mean-subtraction to normalize data from different trials

*Optimizer*: Adam; *Loss function*: CrossEntropyLoss

**Supplementary Table 4**

LAYERS AND TRAINING HYPER-PARAMETERS OF CNN-LSTM

| **Layer** | **Hyper-parameters** |
| --- | --- |
| Conv1d | filter_num=20, filter_size=12, stride=2 |
| Batchnorm | -- |
| Activation | ReLu |
| Dropout | p=0.5 |
| MaxPool | pool_size=2, stride=2 |
| LSTM | unit_num=30, return_sequences=True |
| Batchnorm | -- |
| Activation | tanh(output), sigmoid(recurrent) |
| Dropout | p=0.5 |
| LSTM | unit_num=20, return_sequences=True |
| Batchnorm | -- |
| Activation | tanh(output), sigmoid(recurrent) |
| Dropout | p=0.5, recurrent_p=0.5 |
| Flattern | -- |
| Linear | num_unit=4 |
| Activation | Softmax |

1. Shuffling with the same random seed to ban the impact of data recording order
2. Mean-subtraction to normalize data from different trials

*Optimizer*: Adam; *Loss function*: CrossEntropyLoss; *Learning Rate:* ${10}^{-3}$
